# Supplementary material for: DiscoSnp-RAD: de novo detection of small variants for RAD-Seq population genomics
Source: PeerJ. 2020 Jun 10;8:e9291. doi: 10.7717/peerj.9291 (PMC7293188; doi:10.7717/peerj.9291)
Supplement: Supplemental Information 1 [file peerj-08-9291-s001.pdf]

DiscoSnp-RAD: de novo detection of small variants for population genomics

Jérémy Gauthier[1], Charlotte Mouden[1,2], Tomasz Suchan[3], Nadir Alvarez[4,5], Nils Arrigo[4], Chloé Riou[1], Claire Lemaitre[1], and Pierre Peterlongo[1]

[1]Univ Rennes, Inria, CNRS, IRISA, F-35000 Rennes, France
[2]INRA, BIOGECO, UMR1202, Cestas, France
[3]W. Szafer Institute of Botany, Polish Academy of Sciences, Kraków, Poland
[4]Department of Ecology and Evolution, University of Lausanne, Switzerland
[5]Natural History Museum of Geneva, Geneva, Switzerland

Corresponding author:
Pierre Peterlongo

Email address: pierre.peterlongo@inria.fr

ABSTRACT

Supplementary materials

Supplementary Table 1. *DiscoSnp-RAD* Computational resources on simulated data

# individuals	Wallclock Time (HH:MM:SS)	Memory Peak (GB)	Disk Peak (GB)
100	02:45:24	7.79	28.98
200	06:05:19	14.01	57.69
300	09:27:53	20.52	86.54
400	12:19:17	27.35	115.39
500	16:05:51	34.36	144.24
1000	41:36:29	71.84	288.49

Supplementary Table 2. Comparison of the number of loci identified by each tool

#	# output loci		
individuals	*DiscoSnp-RAD*	*IPyRAD*	*STACKS*
100	77,411	78,395	80,898
200	77,362	78,298	81,955
300	77,335	78,288	81,955
400	77,324	78,276	82,069
500	77,310	78,260	82,151
1000	77,264	78,193	82,511

Supplementary Algorithm 2 (making use of Supplementary Algorithm 1) presents the detailed view of the SNPs and indels detection, including the symmetrical bubble detection and multiple SNPs per bubble.

Supplementary Algorithm 1 $bubble\_extension(path_1, path_2, nb\_sym\_branching, nb\_snps)$

$kmer_1$ = last k nucleotides of $path_1$
$kmer_2$ = last k nucleotides of $path_2$
if $kmer_1$ equals $kmer_2$ **then**
  Output $path_1$,$path_2$
  return
e_1 = set of last character of each children of $kmer_1$ ($e_1 \in \{A,C,G,T\}$)
e_2 = set of last character of each children of $kmer_2$ ($e_2 \in \{A,C,G,T\}$)
if $|e_1| = 0$ and $|e_2| = 0$ **then**
  if last 3 characters from $kmer_1$ and $kmer_2$ are equal **then**
    Output $path_1$,$path_2$
    return
if $|e_1 \cap e_2| = 1$ **then**
  $bubble\_extension(path_1 + e_1 \cap e_2, path_2 + e_1 \cap e_2, nb\_sym\_branching, nb\_snps)$
  return
if $|e_1 \cap e_2| > 1$ **then**
  if $nb\_sym\_branching + 1 \geq max\_branching\_nodes$ **then**
    return
  for All $\alpha \in e_1 \cap e_2$ **do**
    $bubble\_extension(path_1 + \alpha, path_2 + \alpha, nb\_sym\_branching + 1, nb\_snps)$
  return
if $|e_1| = 1$ and $|e_2| = 1$ (thus $e1 \neq e2$) **then**
  if $nb\_snps + 1 \geq max\_snps$ **then**
    return
  $bubble\_extension(path_1 + e_1, path_2 + e_2, nb\_sym\_branching, nb\_snps + 1)$
  return

Supplementary Algorithm 2 *DiscoSnp-RAD* bubble detection for SNPs and indels detection

Create a de Bruijn Graph from all (any number ≥ 1) read set(s)
for Each right branching k-mer in the graph *start* **do**
  for each couple of successor $kmer_1, kmer_2$ of k-mer *start* **do**
    //Snp detection
    $bubble\_extension(kmer_1, kmer_2, 0, 1)$
    //Indel detection from $kmer_1$
    for d in [1, max_index_size] **do**
      Extend $kmer_1$ with d nucleotides
      if last nucleotide of $kmer_1$ equals last nucleotide of $kmer_2$ **then**
        $bubble\_extension(kmer_1, kmer_2, 0, 1)$ with $max\_snps = 1$ (no close SNP with indels)
    //Indel detection from $kmer_2$
    for d in [1, max_index_size] **do**
      Extend $kmer_2$ with d nucleotides
      if last nucleotide of $kmer_2$ equals last nucleotide of $kmer_1$ **then**
        $bubble\_extension(kmer_1, kmer_2, 0, 1)$ with $max\_snps = 1$ (no close SNP with indels)

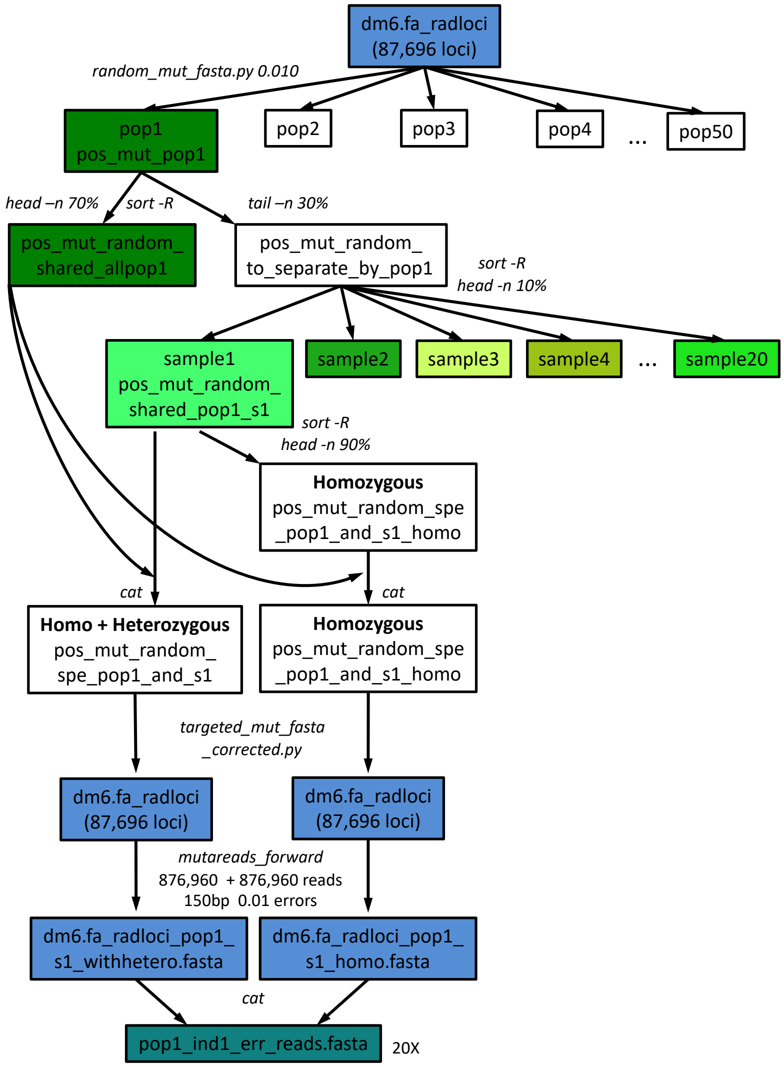

Supplementary Figure 1. Schematic representation of the pipeline designed and used to simulate RAD-Seq data from *D. melanogaster* genome. This pipeline includes RAD-Seq loci extraction, mutation simulation for various samples and populations and Illumina sequencing simulation.

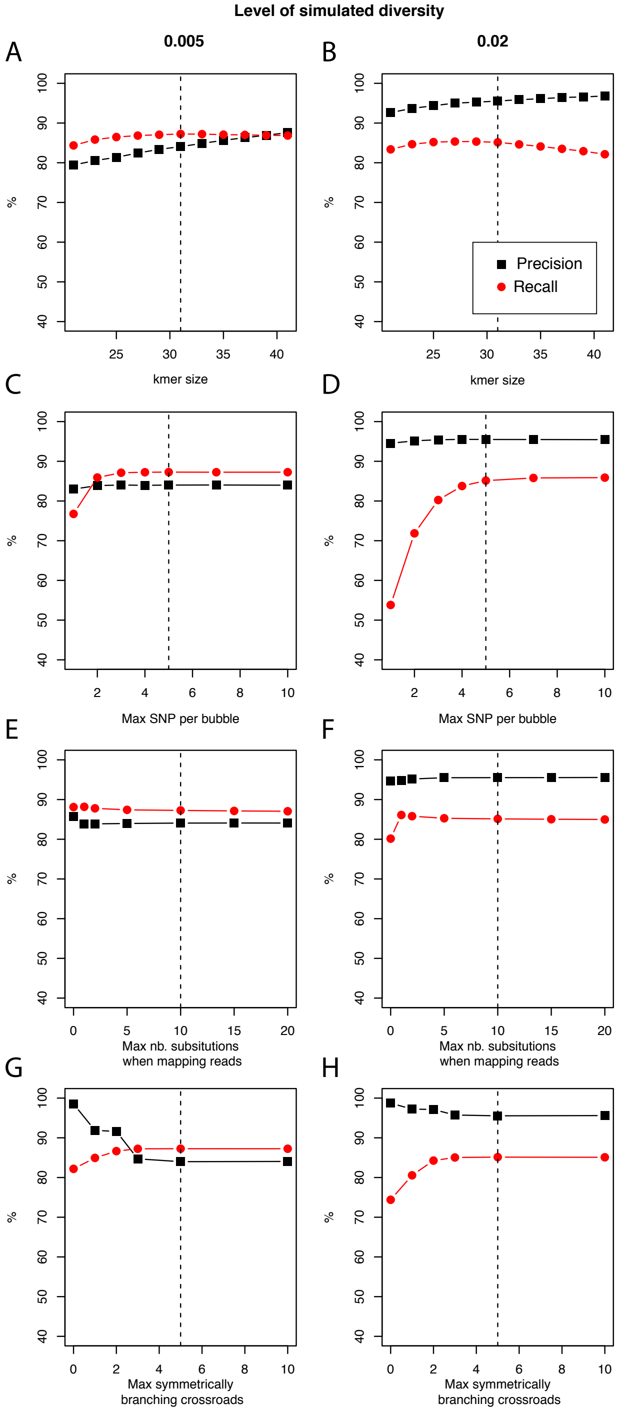

Supplementary Figure 2. Recall and precision on simulated data of 100 samples with two levels of simulated diversity (0.5% and 2%) using *DiscoSnp-RAD* with respect to **A. B.** *k*-mer sizes, **C. D.** maximal number of authorized SNP per bubble, **E. F.** maximal number of authorized substitutions while mapping reads on predicted variants sequences, and **G. H.** maximal number of symmetrically branching crossroads. Dashed vertical line represents on each plot the chosen default value.

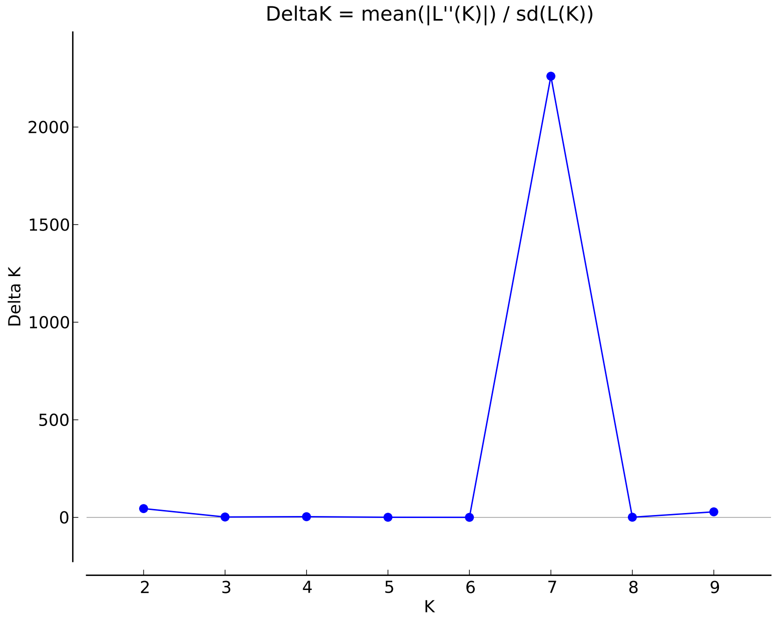

Supplementary Figure 3. Graph of the DeltaK distribution for all tested K, i.e. number of clusters.

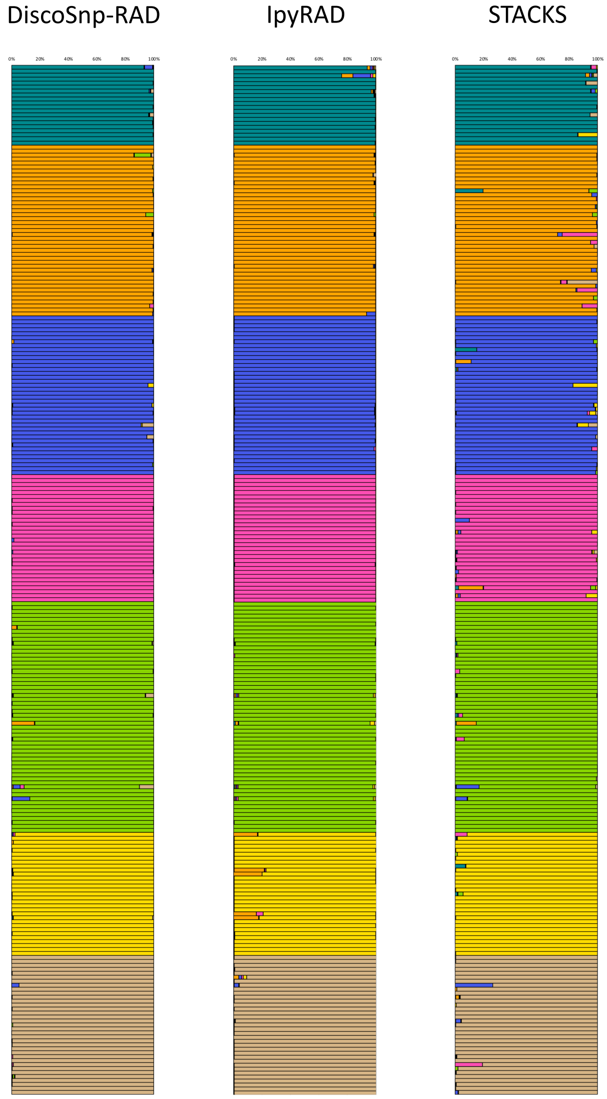

Supplementary Figure 4. Comparison of STRUCTURE assignations for K=7 using SNPs obtained with *DiscoSnp-RAD*, *IPyRAD* and *STACKS* on all samples from the seven *Chiastocheta* species.
